# Productive Replication of HIV-1 but Not SIVmac in Small Ruminant Cells

**DOI:** 10.3390/pathogens11070799

**Published:** 2022-07-15

**Authors:** Hibet Errahmane Chergui, Takfarinas Idres, Chloé Chaudesaigues, Diana Noueihed, Jean Gagnon, Yahia Chebloune

**Affiliations:** 1 PAVAL Laboratory INRAE/UGA USC1450, NanoBio2, Domaine Universitaire, 38400 St Martin d’Hères, France; hibet-errahmane.chergui@univ-grenoble-alpes.fr (H.E.C.); idres.ensv@gmail.com (T.I.); chloe.chaudesaigues@univ-grenoble-alpes.fr (C.C.); diananoueihed7@gmail.com (D.N.); jgagnon@fastmail.com (J.G.); 2 AIOVA NanoBio2 Company, Domaine Universitaire, 38400 St Martin d’Hères, France

**Keywords:** HIV-1, SIVmac, small ruminant cells, restriction, replication

## Abstract

Animal lentiviruses (LVs) have been proven to have the capacity to cross the species barrier, to adapt in the new hosts, and to increase their pathogenesis, therefore leading to the emergence of threatening diseases. However, their potential for widespread diffusion is limited by restrictive cellular factors that block viral replication in the cells of many species. In previous studies, we demonstrated that the restriction of CAEV infection of sheep choroid plexus cells was due to aberrant post-translation cleavage of the CAEV Env gp170 precursor. Later, we showed that the lack of specific receptor(s) for caprine encephalitis arthritis virus (CAEV) on the surface of human cells was the only barrier to their infection. Here, we examined whether small ruminant (SR) cells can support the replication of primate LVs. Three sheep and goat cell lines were inoculated with cell-free HIV-1 and SIVmac viral stocks or transfected with infectious molecular clone DNAs of these viruses. The two recombinant lentiviral clones contained the green fluorescent protein (GFP) reporter sequence. Infection was detected by GFP expression in target cells, and the infectious virus produced and released in the culture medium of treated cells was detected using the indicator TZM-bl cell line. Pseudotyped HIV-GFP and SIV-GFP with vesicular stomatitis virus G glycoprotein (VSV-G) allowed the cell receptors to be overcome for virus entry to further evaluate the viral replication/restriction in SR cells. As expected, neither HIV nor SIV viruses infected any of the SR cells. In contrast, the transfection of plasmid DNAs of the infectious molecular clones of both viruses in SR cells produced high titers of infectious viruses for human indicators, but not SR cell lines. Surprisingly, SR cells inoculated with HIV-GFP/VSV-G, but not SIV-GFP/VSV-G, expressed the GFP and produced a virus that efficiently infected the human indictor, but not the SR cells. Collectively, these data provide a demonstration of the lack of replication of the SIVmac genome in SR cells, while, in contrast, there was no restriction on the replication of the IV-1 genome in these cells. However, because of the lack of functional receptors to SIVmac and HIV-1 at the surface of SR cells, there is specific lentiviral entry.

## 1. Introduction

Animal reservoirs are often the origin of pathogen spillovers that potentially lead to the emergence or re-emergence of several infectious diseases in other hosts, including humans. This cross-species transmission was not only observed recently with the coronavirus disease 2019 (COVID-19) pandemic [1], but LVs were shown to spillover between animal species and zoonosis [2]. They were also the origin of the acquired immunodeficiency syndrome (AIDS) pandemic that started four decades ago [1,3]. Indeed, humans are not the natural hosts of human immunodeficiency virus type 1 (HIV-1); rather, these LVs resulted from multiple zoonotic infections of simian immunodeficiency viruses (SIV) primarily originating from African non-human primates [4,5]. Other instances of SIV jumping the species barrier have been documented between non-human primates (NHP) [6,7,8]. Similarly, it is well accepted now that there is no natural barrier of goat and sheep lentiviruses in small ruminants [9]. Goat lentiviruses were shown to naturally infect sheep [10] and vice versa [11]. These viruses were also shown to cause infections in wild small ruminants [12]. Experimentally infected cows with CAEV do replicate the virus productively up to four months post-infection and then fully clear the virus infection [13,14]. In contrast, moufflons experimentally infected with the same virus remain persistently infected for over two years [15]. Lentivirus transmission is achieved through close contact with blood, semen or milk containing the infected cells [16,17]. However, there are several barriers established by the host at various stages of the infection that need to be bypassed for the virus to replicate effectively in a host; these barriers are referred to as restriction factors. An initial obstacle is the lack of a suitable cell receptor and co-receptors for the virus to bind to, allowing viral entry [18]. In addition, it has been observed that other factors are able to hinder membrane fusion, viral replication, assembly and release. Some of the major restriction factors that have been identified are the apolipoprotein B mRNA editing enzyme catalytic polypeptide 3 (APOBEC3 or A3) family of proteins. These potent host antiviral proteins are able to insert G-to-A hypermutations in the newly synthesized viral genome by their cytidine deaminase activity, in addition to blocking reverse transcription [19,20]. While the HIV-1 Vif accessory protein was found to inhibit APOBEC3G activity in human cells to allow productive virus replication, it fails to counteract APOBEC3G in monkey cells such as rhesus macaques and African monkeys [21]. A single amino acid change between human and macaque APOBOEC3G was shown to be responsible for the inability of HIV-1 Vif to counteract macaque APOBEC3G [22,23,24]. Other studies have further shown that TRIM5α, a cellular protein belonging to the tripartite motif-containing (TRIM) family of proteins, represses HIV-1 from infecting Old World monkey cells, in addition to other retroviruses in multiple species, by destabilizing capsid uncoating. This protein recognizes and degrades the capsid protein of retroviruses, thereby cancelling their replication in target cells [25,26]. However, although a wide variety of species have evolved several restriction factors as a part of their line of defense as innate immunity against viral infections, including lentiviruses, these latter ones have adapted by using a variety of strategies to bypass either one or many of these restriction factors. Indeed, the specific cell receptors can be bypassed either by using an alternative receptor, or by using an entry mechanism that does not depend on the presence of a receptor [18,27]. To counter APOBEC3, lentiviruses have also developed, through their viral infectivity factor (Vif) accessory protein, the capacity to degrade these APOBEC3 proteins [19,20]. Along with that, viral capsid mutations can help escape the binding to TRIM proteins [28]. Some studies have also reported that tetherin (BST-2) inhibits the release of mature viral particles from the cell surface; nonetheless, it is antagonized by the viral protein U (Vpu) [29]. Nef and Env proteins were also reported to antagonize tetherin [30,31]. The CD225 superfamily comprises the human interferon-induced transmembrane (IFITMs) family of proteins that inhibit a broad range of viruses by interfering with viral-to-cellular membrane fusion (reviewed in Bailey CC, Zhong G, Huang I-C, Farzan M. 2014. IFITM-family proteins: the cell’s first line of antiviral defense. Annu Rev Virol 1:261–283. https://doi.org/10.1146/annurev-virology-031413-085537, accessed on 24 June 2022). IFITM1 was shown to downregulate HIV-1 p24 expression in infected cells, resulting in a reduction in viral production. To circumvent IFITM1, a study demonstrated that HIV-1 acquires mutations located in its Vpu and Env proteins promoting viral propagation between cells, while IFITM2 and IFITM3 are believed to interfere with endocytosis-mediated HIV-1 entry and trafficking [32,33]. Other examples of restriction factors include cholesterol-25-hydroxylase (CH25H) and serine incorporator 5 (SERINC5) that disrupt virus–cell fusion and γ-IFN-inducible lysosomal thiolreductase (GILT) that alters viral glycoprotein activation affecting viral entry as well. However, as for the other host restriction factors, counteracting machinery was established by HIV-1 [34,35,36]. The most recently identified restriction factor is SAMHD1, a deoxynucleoside triphosphate triphosphohydrolase believed to hydrolyze most cellular dNTPs, thus inhibiting reverse transcription and viral complementary DNA (cDNA) synthesis [37]. In parallel, it was also demonstrated that the Vpx proteins from some retroviruses render cells that are usually retractive to HIV-1 (human dendritic, myeloid lineage and resting CD4+ T cells), permissive to this latter infection through proteasomal degradation of SAMHD1 [38,39]. 

We have demonstrated, in a previous study, that the lack of a specific receptor for caprine encephalitis arthritis virus (CAEV) on the surface of human cells is the only barrier that protects human cells from this lentivirus infection [40]. In another study, we showed that although CAEV caused infection in ovine fibroblasts (sheep choroid plexus cells), the virus life cycle was terminated prior to productive replication. This inability of CAEV to replicate in these cells was found to correlate with aberrant proteolytic processing of the Env glycoprotein, indicating the existence of another type of restriction [41]. One of the main objectives of our lab is to better understand the mechanisms behind cross-species infections, adaptation and the increase in the virulence of pathogens. In this context, an observation made in Japan that reported attempted sexual interactions between Japanese macaques and wild deer is one of the reasons that prompted us to conduct this study [41]. We explored whether SR cell lines are permissive to primate lentiviruses, SIVmac and HIV-1, and if not, what level the restriction of infection is based.

## 2. Results

### 2.1. pSIV-GFP and pHIV-GFP DNA Plasmids Successfully Express Viral Genomes in All Transfected SR Cell Lines

**Figure 1:**
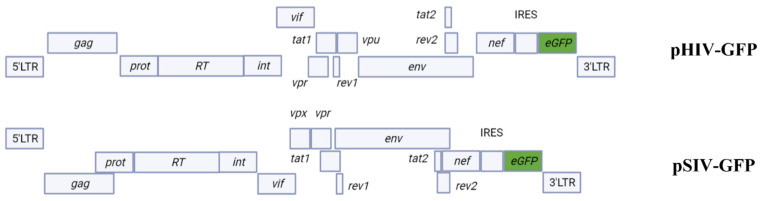
Organization of pHIV-GFP and pSIV-GFP plasmid DNAs genomes.

We first wanted to examine whether SIVmac and HIV-1 genomes are expressed in cell lines originating from different species. These cell lines were transfected with pHIV-GFP or pSIV-GFP plasmid DNAs (Figure 1), as described in the Materials and Methods section.

**Figure 2:**
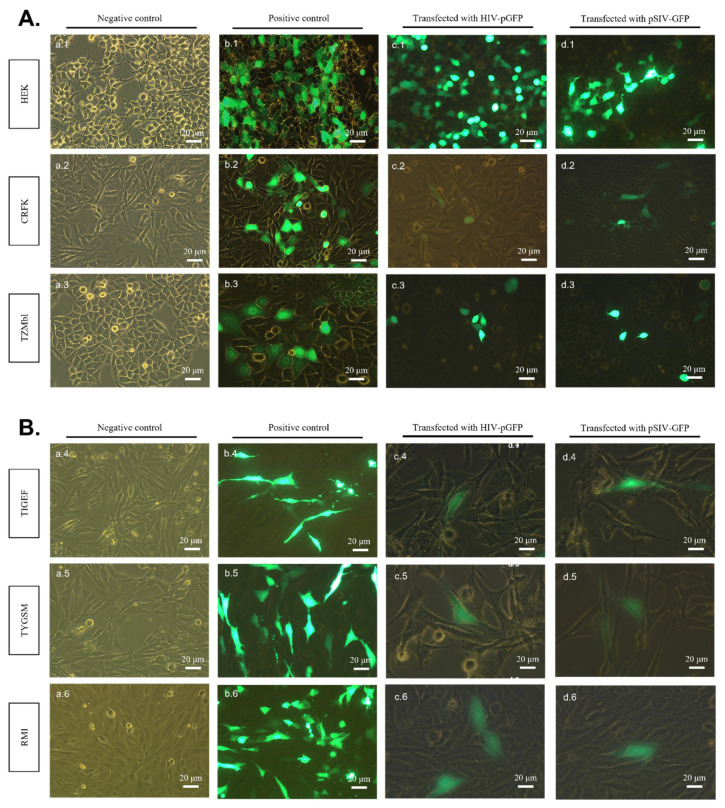
Detection of GFP expression in pHIV-GFP and pSIV-GFP transfected cells. (**A**) HEK, CRFK and TZM-bl cell lines. (**B**) TIGEF, TYGSM and RMI cell lines. Cell monolayers were transfected as described in Materials and Methods. At 24 h post-transfection, the cell monolayers were observed under a fluorescence microscope to assess the expression of GFP. (c.1–c.6) Cells transfected with pHIV-GFP. (d.1–d.6) Cells transfected with pSIV-GFP. (a.1–a.6) Non-transfected cells were used as a negative control. (c.3) TZM-bl cells transfected with pHIV-GFP and (d.3) pSIV-GFP were used as a positive control along with cells transfected with GFP plasmid (b.1–b.6). The images were acquired as a merge of the green channel and the bright field. Acquisitions were performed with 488 nm excitation and the emission was collected at 500–600 nm.

An examination of cells by fluorescent microscopy at 24 h post-transfection revealed GFP expression in all transfected cell lines with both pHIV-GFP and pSIV-GFP DNAs. No GFP expression was detected in any of the non-transfected control cells (Figure 2A,B). Indeed, green fluorescence was noted in HEK and CRFK cells transfected with pHIV-GFP and pSIV-GFP DNAs (Figure 2A; c.1, c.2, d.1, d.2), as well as TIGEF, TYGSM and RMI cells (Figure 2B; c.4–c.6, d.4–d.6). The proportions of GFP-expressing cells in each sample were examined by flow cytometry and are reported in Supplementary Figure S1.

### 2.2. SIV-GFP and HIV-GFP Viruses Cannot Directly Infect SR Cell Lines

**Figure 3:**
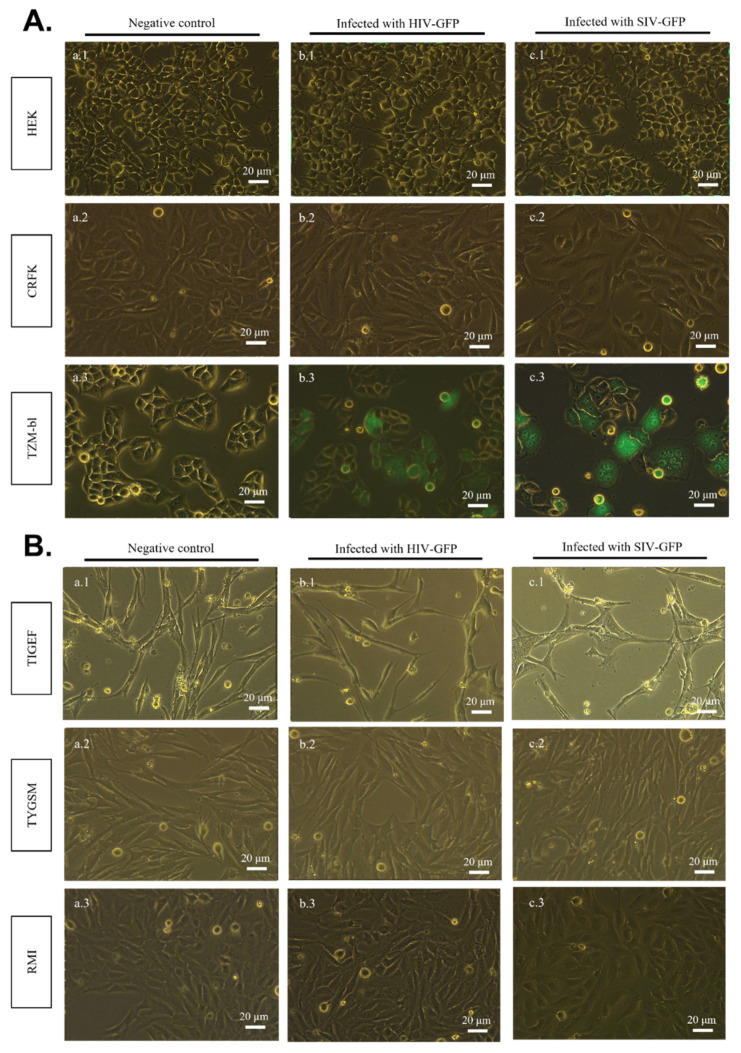
Detection of SIV-GFP and HIV-GFP infection by fluorescence microscopy. (**A**) HEK, CRFK and TZM-bl human and feline cell lines. (**B**) TIGEF, TYGSM and RMI SR cell lines. Monolayers of each of the cell lines were inoculated with HIV-1 and SIVmac viral stocks expressing GFP as indicated in Materials and Methods. At 120 h post-infection, the monolayers were observed under a fluorescence microscope to assess the expression of GFP. (b.1–b.3) Cells inoculated with HIV-GFP. (c.1–c.3) Cells inoculated with SIV-GFP. (a.1–a.3) Non-inoculated cell lines were used as a negative control. (b.3) TZM-bl cells inoculated with SIV-GFP and (c.3) HIV-GFP were used as positive controls. The images are a merge of the green channel and the bright field. They were acquired with 488 nm excitation and the emission was collected at 500–600 nm.

To evaluate whether HIV-1 and SIVmac can enter and replicate in the studied cell lines, infectious HIV-GFP and SIV-GFP particles were produced in HEK cells, harvested, and were used to inoculate HEK, CRFK, TYGSM, TIGEF and RMI cells. As TZM-bl cells express the receptors and co-receptors for HIV and SIV, and are permissive to these virus replications, as mentioned before, they were used as a positive control for infection. Non-inoculated cells of each cell line were used as negative controls. Five days after the viral inoculation, the GFP expression was assessed in all the cell lines (Figure 3A,B). The results show that, except TZM-bl cells (Figure 3A; b.3, c.3), none of the other cell lines (Figure 3A,B) were found to be permissive to either HIV-GFP or SIV-GFP infectious particles, since no green fluorescence was detected in any of the cell cultures. The proportions of GFP-expressing cells in each sample were examined by flow cytometry and are reported in Supplementary Figure S2.

### 2.3. SR Cells Are Permissive to HIV-GFP, but Not SIV-GFP, Pseudotyped with VSV-G 

**Figure 4:**
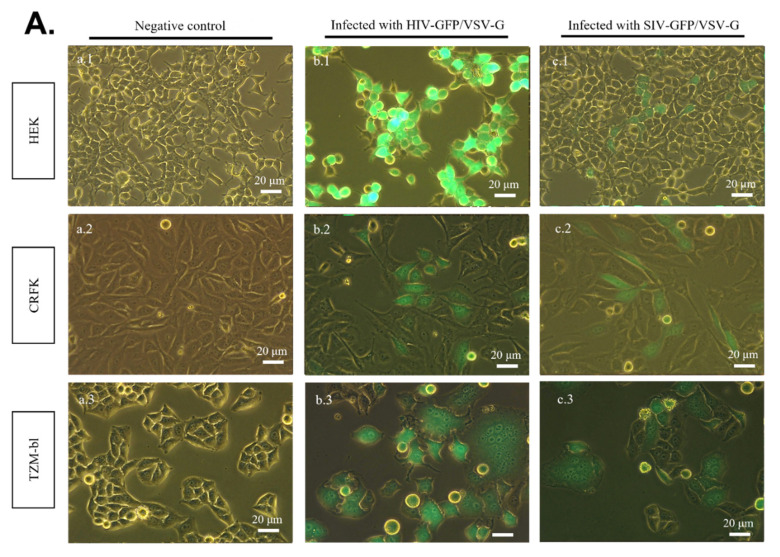

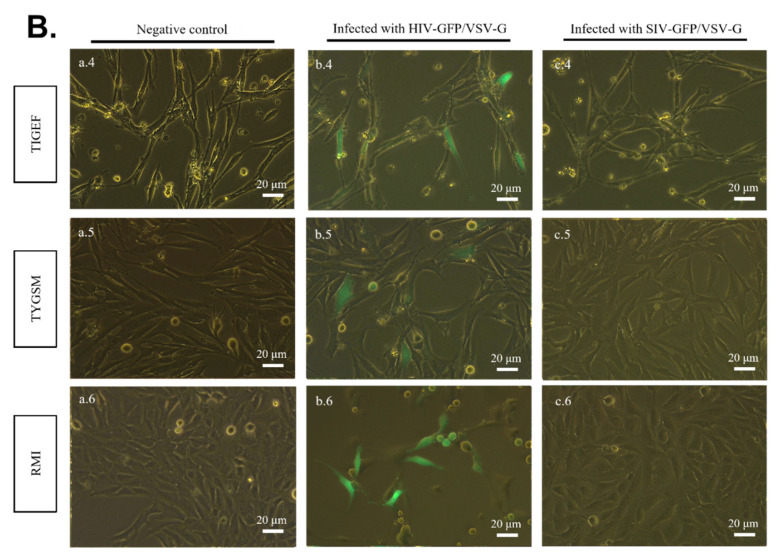
Detection of HIV-GFP/VSV-G and SIV-GFP/VSV-G infection by fluorescence microscopy. (**A**) HEK, CRFK and TZM-bl cell lines. (**B**) TIGEF, TYGSM and RMI cell lines. The cell lines were inoculated with SIV-GFP and HIV-GFP pseudotyped with VSV-G. At 120 h post-inoculation, the cell lines were observed under a fluorescence microscope to assess GFP expression in the monolayers. (b.1–b.6) Cells inoculated with HIV-GFP/VSV-G. (c.1–c.6) Cells inoculated with SIV-GFP/VSV-G. (a.1–a.6) Non-transfected cell lines were used as a negative control. TZM-bl cells inoculated with (b.3) HIV-GFP/VSV-G and (c.3) SIV-GFP/VSV-G were used as a positive control, respectively. The images are a merge of the green channel and the bright field. They were acquired with 488 nm excitation and the emission was collected at 500–600 nm.

To further investigate the productive/restrictive viral replication of HIV-1 and SIVmac in the studied cell lines, the latter were inoculated with VSV-G pseudotyped HIV-GFP and SIV-GFP. VSV-G pseudotyped virions will have the ability to bypass cell membrane receptors whose absence or non-functionality usually constitutes the first restriction factor to viral replication. The results reported in Figure 4 show that 120 h post-inoculation, GFP-positive cells were observed in HEK–293T and CRFK monolayers, demonstrating that these cells were permissible to both VSV-G pseudotyped HIV-GFP and SIV-GFP (Figure 4A; b.1, b.2, c.1, c.2). However, although no green florescence was observed in any of the SR cell lines inoculated with SIV-GFP/VSV-G (Figure 4B; c.4–c.6), GFP-positive cells were observed in all SR cell lines inoculated with HIV-GFP/VSV-G, demonstrating their productive infection (Figure 4B; b.4–b.6).

**Figure 5:**
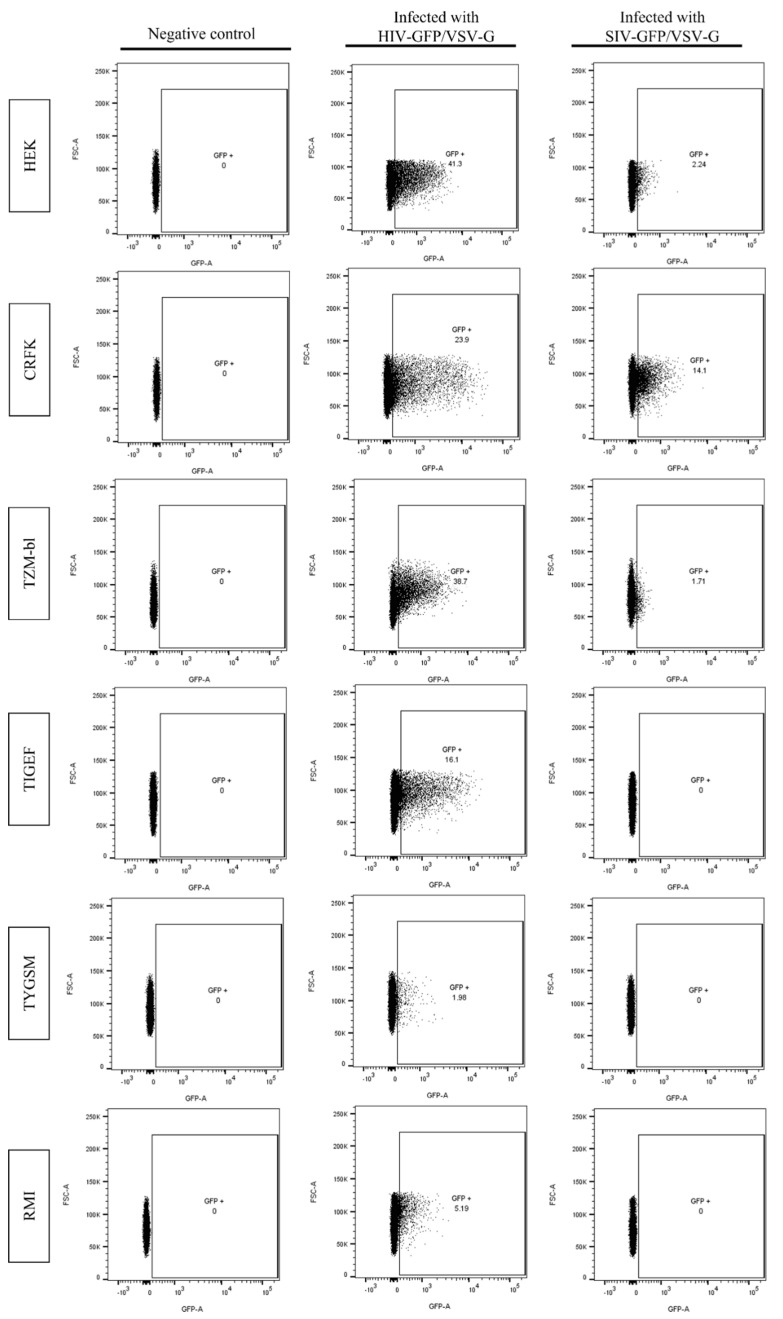
Flow cytometry analyses of cells inoculated with VSV-G pseudotyped HIV-GFP and SIV-GFP. Cells were acquired in a FACSCantoII and displayed according to FSC/GFP characteristics. Cells were analyzed using FlowJo software. Non-inoculated cells were used as negative controls.

To evaluate the proportions of GFP-positive cells in each of the cell lines inoculated with HIV-GFP/VSV-G and SIV-GFP/VSV-G, samples were analyzed by flow cytometry. As shown in Figure 5, all cell lines inoculated with HIV-GFP/VSV-G have 1–38% of GFP-expressing cells. In contrast, no GFP-positive cell was detected in SR cell lines inoculated with SIV-GFP/VSV-G, while the proportion of GFP-positive human and feline cell lines was 1–14% (Figure 5).

## 3. Discussion

In the present study, our objective was to examine whether HIV-1 and SIVmac are restricted or have the potential to replicate in non-primate cells. Our data showed that small ruminant (TYGSM, TIGEF, RMI), feline (CRFK) and human (HEK) cells lacking the appropriate receptors and co-receptors are, as expected, not susceptible to HIV-1 and SIVmac infections. Nonetheless, all of these cell lines productively expressed HIV-1 and SIVmac genomes, together with the GFP transgene marker, when the infectious molecular clone DNAs were introduced into these cells by transfection. These results clearly demonstrated the absence of any restriction factor capable of blocking the late stages of expression of HIV-1 and SIVmac in SR cells, HEK cells and feline cells.

Following bypassing HIV-1- and SIVmac-specific receptors on the surface of target cells by means of VSV-G pseudotyping the virions, intriguing results were obtained. SIV-GFP/VSV-G productively infected CRFK and HEK cells, thereby showing no post-entry restriction of virus replication. In contrast, SIV-GFP/VSV-G failed to show any sign of infection in all three inoculated SR cell lines; no GFP-expressing cells were observed. As for the cells infected with HIV-GFP/VSV-G, although we obtained the same profile in CRFK and HEK cells, interestingly, GFP-positive cells were observed in all three SR cell lines inoculated with HIV-GFP/VSV-G. This demonstrated the absence of any post-entry restriction of HIV-1 replication in all the studied SR cell lines after countering the entry receptors/co-receptors in these cells. This unexpected observation contrasts previous studies in other species, demonstrating that the replication of HIV-1 is strongly restricted in mouse cells, but also in primate macaque cells, independently of receptor/coreceptor usage [42,43]. Indeed, a number of host factors restrictive for HIV-1 have been identified in murine cells. Among these, the absence of a functional receptor/co-receptor for HIV-1 on the surface of murine cells [44], the lack of functional cyclin T1 for post-entry early HIV-1 gene expression [45], and the restriction of HIV-1 replication by mouse APOBEC3G [46]. In contrast, in addition to the lack of a functional receptor for SIVmac on the surface of SR cells, there was post-entry restriction. This restriction occurred in the early stages, since it does not prevent the replication of infectious DNA after transfection. Taken together, these results clearly indicate that the replication of SIVmac, but not HIV-1, is restricted in SR cells in the post-entry compartment. However, although studying host–pathogen interactions in cell culture models is a quick, efficient and economic practice, it does come with some limitations. Modeling complex processes, such as viral infections, in single-lineage monocultures evidently does not reflect all events taking place in vivo. Replicating all the biological networks relevant to this study in an animal model would result in more complex and high-cost experimental designs. Nevertheless, further studies would be interesting to identify, with certainty, the restriction mechanisms of SIVmac in SR cells. These would bring insights into evaluating the risks of bypassing this type of restriction and the emergence of new lentiviruses. Indeed, a detailed understanding of how a virus jumps species boundaries to infect another host will help in the prevention of future zoonotic events.

## 4. Material and Methods

### 4.1. Plasmids 

The HIV-1 pNL4-3 IRES-eGFP (pHIV-GFP) and pSIVmac-eGFP (pSIV-GFP) vector plasmids containing a cytomegalovirus (CMV)-driven enhancer and co-expressing the green fluorescent protein (eGFP) that was inserted downstream of the nef gene were kindly provided by Frank Kirchhoff [47]. The pCS-H2B-eGFP plasmid encodes the eGFP gene under the CMV promoter/enhancer. The pCMV-VSV-G plasmid expresses the G glycoprotein of vesicular stomatitis virus (VSV) under the CMV promoter/enhancer. These two recombinant plasmids were purchased from Addgene Europe, Teddington, UK. JM109 competent bacteria (Promega, Charbonnière, France) were transformed with the various plasmids and were amplified using the classical culture conditions. Plasmids were then extracted and purified using a plasmid extraction and purification kit Macherey-Nagel™(MACHEREY-NAGEL SAS, Hoerdt, France) according to the manufacturer’s instructions.

### 4.2. Cell Culture 

Human embryonic kidney 293 cells (HEK 293T), TZM-bl (HeLa cell derivative engineered to express CD4, CCR5 and CXCR4 on their surface, and to contain integrated reporter genes for firefly Luc and *E. coli* β-galactosidase under the control of an HIV–1 long terminal repeat) [48] were obtained from the NIH AIDS reagent repository. Together with Crandell–Rees Feline Kidney (CRFK) cells, they were grown and maintained in Dulbecco’s modified Eagle’s medium (DMEM), supplemented with 10% fetal bovine serum (FBS), 1% glutamine and 1% penicillin–streptomycin (Eurobio, Les Ulis, France). Goat synovial membrane (TYGSM), goat embryo fibroblasts (TIGEF) and sheep fetal kidney (RMI) are cell lines that were previously immortalized in our laboratory [49,50]. They were cultured in Eagle’s minimum essential medium (MEM) supplemented with 10% FBS, 1% of glutamine and 1% penicillin–streptomycin. All the cell cultures were maintained in a tissue culture incubator at 37 °C, 5% CO_2_ under saturating humidity.

### 4.3. Cell Transfection

The cell lines were seeded into 6-well cell culture plates at a density of 1 × 10^6^ cells/well in 2 ml of medium and were maintained for 24 h at 37 °C, 5% CO_2_ under saturating humidity to achieve ≥ 80% confluency before inoculation with a mixture of plasmid DNA (2.5 μg) and TransIT-LT1 reagent (MIR2300, Euromedex France, Souffelwyersheim, France), according to the manufacturer’s recommendations. Non-transfected cells and cells transfected with a plasmid expressing pCS-H2B-eGFP were used as negative and positive controls, respectively. After incubation at 37 °C, 5% CO_2_ and under saturated humidity for 24 h, cell monolayers were rinsed twice with 1 × PBS and then 2 mL of fresh cell medium was added. Cells expressing GFP were then observed under a fluorescent microscope to assess the efficiency of the transfection and examine the expression of HIV-GFP and SIV-GFP genomes. DNA transfection experiments and observations were reproduced at least three times. 

### 4.4. Production of Viral Stocks 

To generate SIV-GFP/VSV-G and HIV-GFP/VSV-G virus stocks, monolayers of HEK cells were co-transfected with pCMV-VSV-G with either pSIV-GFP or pHIV-GFP plasmids in a ratio 2:1. After incubation at 37 °C, 5% CO_2_ and under saturated humidity for 18 h, the transfection medium was discarded, and cell monolayers were rinsed twice with 1 × PBS and then 2 mL of fresh cell medium was added. HIV-GFP and SIV-GFP stocks were produced similarly, except without co-transfection with the VSV-G plasmid. At 48 h post-transfection, supernatants were harvested, cleared by filtration through 0.45 μm filters and stored at –80 °C as aliquot fractions until use. The titers of VSV-G pseudotyped virus stock were determined by inoculating HEK-293T and TYGSM cells with serial dilutions and scoring GFP-expressing cells under a fluorescence microscope. Virus productions were reproduced three times. 

### 4.5. Infection of Cell Lines

To examine the susceptibility of the studied cell lines to virus infections, HEK–293T, TYGSM, TIGEF, RMI, CRFK monolayer cells seeded into 12-well plates were inoculated at MOI = 0.1 with 10^4^ GFP forming units of SIV-GFP and HIV-GFP or the pseudotyped SIV-GFP/VSV-G and HIV-GFP/VSV-G virus stocks. The TZM-bl cell line was used as a positive control. Cells inoculated with supernatant collected from non-transfected cells were used as a negative control. After 18 h incubation at 37 °C, 5% CO_2_ and under saturated humidity, cell monolayers were rinsed twice with 1 × PBS and replenished with 2 mL of fresh cell medium. Transduction efficiency was examined at 5 days post-inoculation under fluorescent microscope. This experiment was repeated at least three times.

### 4.6. Flow Cytometry Analysis

Cell samples were detached following trypsin treatment and fixed with paraformaldehyde 4%. Cells were acquired using BD FACSCanto II, according to the SSC/FSC. GFP fluorescence was detected following excitation with an argon ion laser source at 488 nm. The percentage of GFP-positive cells was determined in comparison to the negative control. The data analysis was performed using FlowJo v10 software (BD Biosciences, Le Pont de Claix, France). For each sample, a minimum of 10,000 events were collected.

## Data Availability

Not applicable.

## Supplementary Materials

The following supporting information can be downloaded at: https://www.mdpi.com/article/10.3390/pathogens11070799/s1, Figure S1. Flow cytometry analyses of cells transfected with pHIV-GFP and pSIV-GFP. Cells were acquired in a FACSCantoII and displayed according to FSC/GFP characteristics. Cells were analyzed using a FlowJo software. Non inoculated cells were used as negative controls. Figure S2. Flow cytometry analyses of cells inoculated with HIV-GFP and SIV-GFP. Cells were acquired in a FACSCantoII and displayed according to FSC/GFP characteristics. Cells were analyzed using a FlowJo software. Non inoculated cells were used as negative controls.
