# Supplementary material for: Productive Replication of HIV-1 but Not SIVmac in Small Ruminant Cells
Source: Pathogens. 2022 Jul 15;11(7):799. doi: 10.3390/pathogens11070799 (PMC9316499; doi:10.3390/pathogens11070799)
Supplement: Supplementary file 1 [file pathogens-11-00799-s001.zip › pathogens-1709305-supplementary.pdf]

Supplementary data

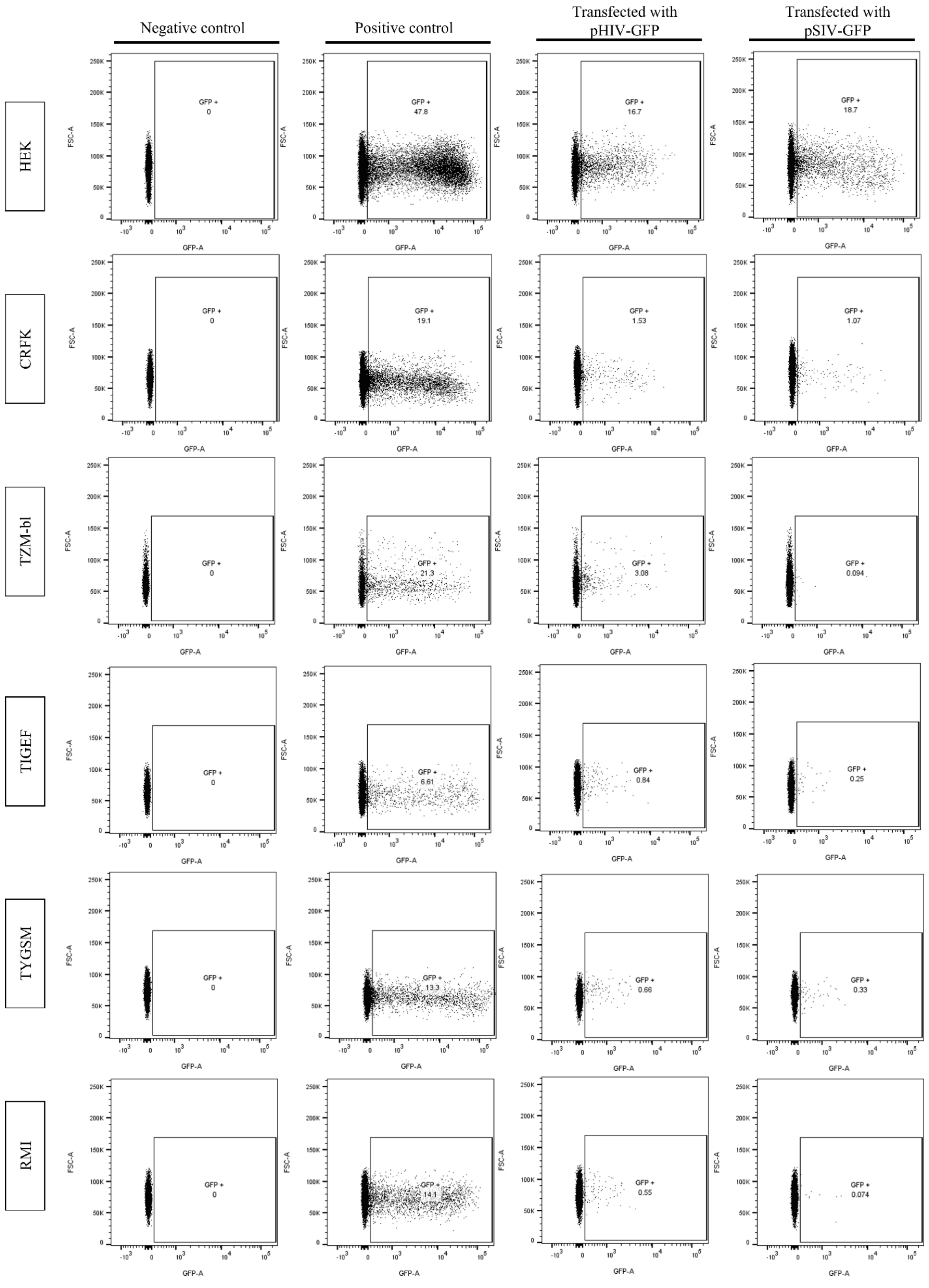

Figure S1. Flow cytometry analyses of cells transfected with pHIV-GFP and pSIV-GFP. Cells were acquired in a FACSCantoII and displayed according to FSC/GFP characteristics. Cells were analyzed using a FlowJo software. Non inoculated cells were used as negative controls.

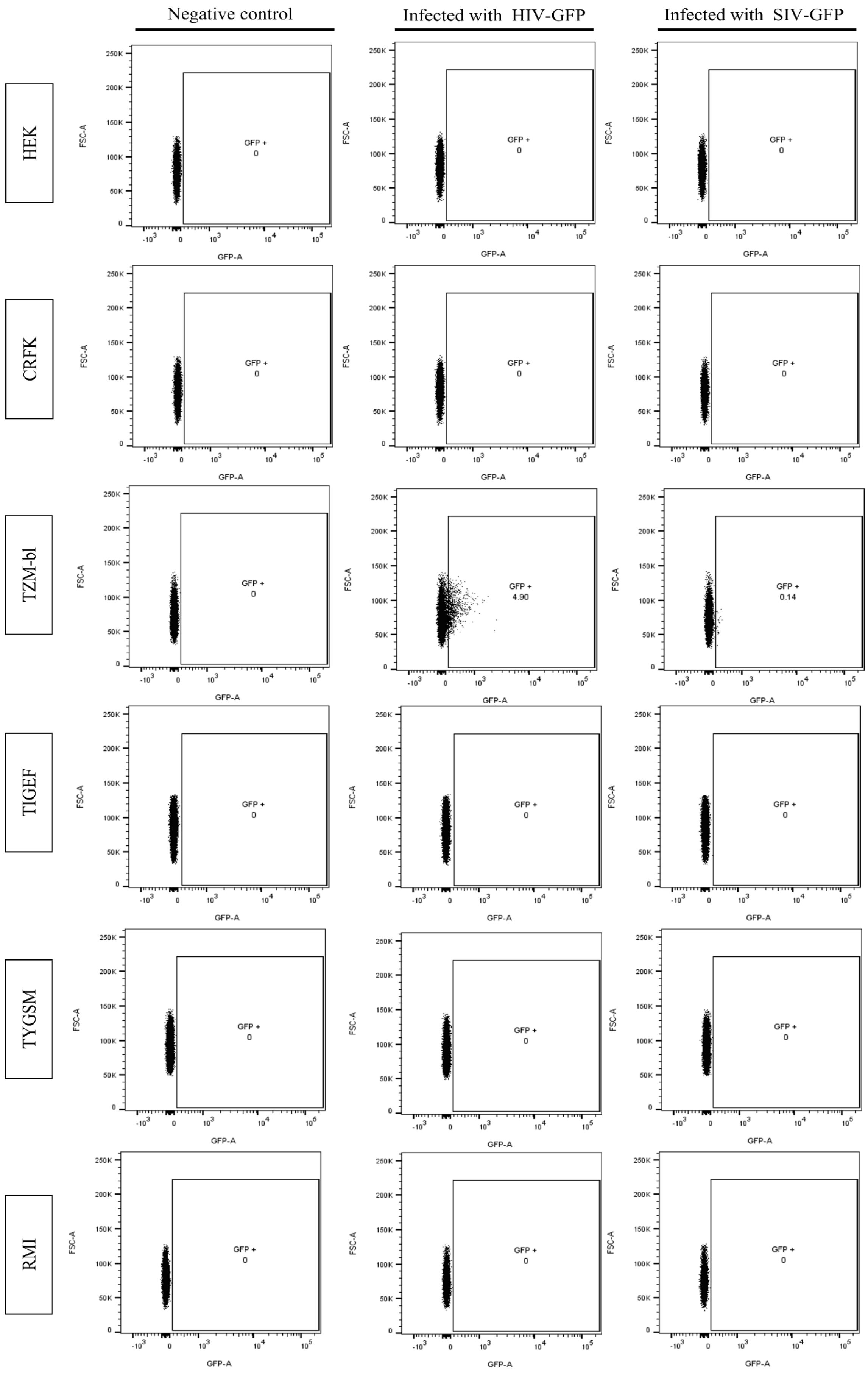

Figure S2. Flow cytometry analyses of cells inoculated with HIV-GFP and SIV-GFP. Cells were acquired 10 in a FACSCantoII and displayed according to FSC/GFP characteristics. Cells were analyzed using a FlowJo software. Non inoculated cells were used as negative controls.
